# Animal Teaching

**Published:** 1874-04

**Authors:** 


### ANIMAL TEACHING.

LONG years before the American Rarey's name was heard as a horse tamer a secret existed, as a family heirloom, among a branch of the O'Sullivans in the south of Ireland. This family was known as "The Whisperers," and they possessed the power of rendering as quiet as a lamb the most stubborn and unmanageable horse that ever existed. Whether they did anything more to a horse than breathe into his nostrils we know not, but by doing this, and by kindness, soothing, and other ways known to themselves, they effected their purpose and retained their fame. Putting the question of drugs, or stimulants, or other fascinating means aside, and coming to the point of pure and unadulterated domestication and teaching, perhaps there was no person in modern times achieved so much success in animal teaching as S. Bisset. This man was an humble shoemaker. He was born in Scotland in 1721, but he afterwards removed to London, where he married a woman who brought him some property. Then, turning to a broker, he accumulated money until the year 1759, when his attention was turned to the training of animals, birds and fishes. He was led into this new study on reading an account of a remarkable horse shown at a fair at St. Germain.

Bissett bought a horse and a dog, and succeeded beyond his expectations in teaching them to perform various feats. He next purchased two monkeys, which he taught to dance and tumble on a rope, and one would hold a candle in one paw and turn a barrel organ with the other while his companion danced. He next taught three cats to do a great many wonderful things, to sit before music books and to squall notes pitched to different keys. He advertised a "cat's opera" in the Haymarket and successfully carried out his programme, the cats accurately fulfilling their parts. He pocketed some thousands by these performances. He next taught a leveret, and then several species of birds to spell the name of any person in the company, and to distinguish the hour of the day or night. Six turkey cocks were next rendered amenable to a country dance, and after six months' teaching he trained a turtle to fetch and carry like a dog, and having chalked the floor and blackened its claws, made it trace out the name of any given person in the company.—*Land and Water.*

